# Backbone chemical shift assignment and dynamics of the N-terminal domain of ClpB from *Francisella tularensis* type VI secretion system

**DOI:** 10.1007/s12104-021-10062-3

**Published:** 2022-01-05

**Authors:** Ameeq Ul Mushtaq, Jörgen Ådén, Athar Alam, Anders Sjöstedt, Gerhard Gröbner

**Affiliations:** 1 Department of Chemistry, University of Umeå, 901 87 Umeå, Sweden; 2 Department of Clinical Microbiology, University of Umeå, 901 87 Umeå, Sweden

**Keywords:** NMR resonance assignment, ^15^N relaxation, ClpB chaperone, Type VI secretion system, *Francisella tularensis*

## Abstract

The Hsp100 family member ClpB is a protein disaggregase which solubilizes and reactivates stress-induced protein aggregates in cooperation with the DnaK/Hsp70 chaperone system. In the pathogenic bacterium *Francisella tularensis,* ClpB is involved in type VI secretion system (T6SS) disassembly through depolymerization of the IglA-IglB sheath. This leads to recycling and reassembly of T6SS components and this process is essential for the virulence of the bacterium. Here we report the backbone chemical shift assignments and ^15^N relaxation-based backbone dynamics of the N-terminal substrate-binding domain of ClpB (1-156).

**Supplementary Information:**

The online version contains supplementary material available at 10.1007/s12104-021-10062-3.

## Biological context

**Fig. 1:**
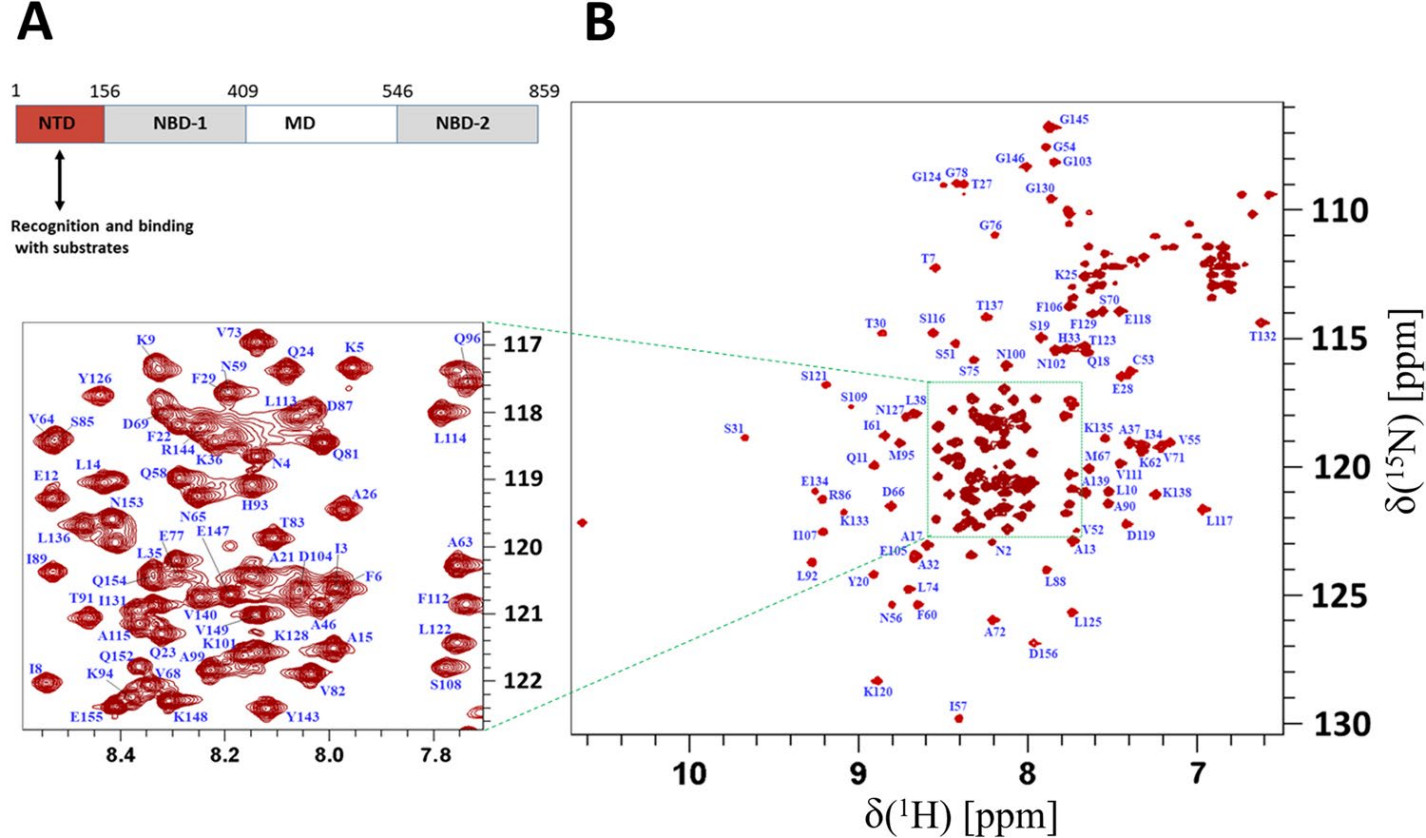
**A** Schematic picture showing the domain organization of ClpB from *F. tularensis,* comprising of four domains: N-terminal domain (NTD), two nucleotide-binding domains (NBD-1 and NBD-2), and middle domain (MD). **B** ^1^H-^15^N HSQC spectrum of ^15^N/^13^C-labeled 1.7 mM NTD ClpB (1-156) in 20 mM NaPi, 20 mM NaCl (pH 6.5) and 10% (v/v) D_2_O buffer showing the assignment of backbone amides indicated with one-letter codes for amino acids and the residue numbers in blue. All spectra were acquired at 310 K at 850 MHz ^1^H frequency

ClpB is a member of ring-forming AAA + family ATPases that cooperates together with the DnaK/Hsp70 system to reactivate stress-denatured aggregated proteins. ClpB, like other Hsp100 family members, has a homohexameric structure. The monomer of ClpB comprises four domains: an N-terminal domain (NTD) important for substrate recognition and binding, the first nucleotide binding domain (NBD-1), the flexible middle domain (MD), and a second nucleotide binding domain (NBD-2) (Alam et al. 2021) as shown in Fig. 1A. The physiological role of ClpB under different conditions has been extensively studied since it provides protection against stress conditions, e.g. heat, low pH, osmotic and oxidative stress, ethanol, and nutrient starvation. ClpB-deficient mutants demonstrate tremendously decreased survival upon exposure to these stresses (Meibom et al. 2008; Krajewska et al. 2017; Tripathi et al. 2020; Glaza et al. 2021). ClpB is therefore critical for survival and infectivity of a broad range of clinically relevant microorganisms.

Besides its role in solubilizing stress-induced protein aggregates, a role of ClpB in type VI secretion (T6S) has recently been reported in the highly pathogenic intracellular bacterium *Francisella tularensis* that infects and replicates mainly inside macrophages and causes the disease tularemia in a large number of mammalian species (Brodmann et al. 2017; Alam et al. 2020). A recent report suggests that ClpB apparently serves as a functional homolog of ClpV and it harnesses energy generated by the hydrolysis of ATPs and it is required for depolymerization of the T6SS sheath and the subsequent recycling and reassembly of the T6SS components. Therefore, the deletion of *clpB* leads to significantly reduced level of T6S and complete attenuation of *F. tularensis* in mice (Alam et al. 2018, 2020).

Functional ClpB orthologs are absent in mammals, including *Homo sapiens*, thus ClpB has the potential to serve as drug target for the development of novel antimicrobials. Since ClpB is an essential factor in bacterial stress response and pathogen virulence, inhibition of ClpB might suppress infectivity and the survival of invading pathogens. Here we present the backbone chemical shift assignments and dynamics of the N-terminal substrate binding domain of ClpB (1-156) that binds to the IglA-IglB sheath which is important for recycling and reassembly, and essential for the virulence of the bacterium, and can therefore be a potential drug target.

## Methods and experiments

### Protein expression and purification of isotope-labelled NTD ClpB(1-156)

The first 156 N-terminal residues of the ClpB chaperone from *F. tularensis* [NTD ClpB (1-156)] were cloned into the pET-His1a expression vector using essentially the same strategy as reported before (Alam et al. 2020). Expression was initiated by transforming 1 µl of plasmid into 100 µl of *Escherichia coli* BL21 (DE3) competent cells, and plated on agar plates with 50 µg/ml kanamycin. The following day, cells were transferred into a pre-culture consisting of 20 ml 1 × LB with 50 µg/ml kanamycin, and further grown at 37 °C overnight. 10 ml of fresh culture was transferred into M9 medium prepared (per liter) as following: 6 g Na_2_HPO_4_, 3 g KH_2_PO_4_, 0.5 g NaCl, 6.25 g glucose, 1 g NH_4_Cl, 11 mg CaCl_2_, 1 g MgSO_4_·7H_2_O, and trace elements (1 ml of 50 mM FeCl_3_, 20 mM CaCl_2_, 10 mM MnCl_2_, 10 mM ZnSO_4_, 2 mM CoCl_2_, 2 mM CuCl_2_, 2 mM NiCl_2_, 2 mM Na_2_MoO_4_ and 2 mM H_3_BO_3_ per liter medium). The pH was adjusted to 7.1 with NaOH and sterile filtrated using a 20 µm sterile filter prior to use. For ^15^N labeling, 1 g of NH_4_Cl was replaced with ^15^NH_4_Cl, and for ^15^N/^13^C labeling, the glucose was also substituted with 1.2 g ^13^C glucose per liter M9 media (both from Cambridge Isotope Laboratories, Inc., Tewksbury, MA, USA). The culture was supplemented with 50 µg/ml kanamycin and grown at 37 °C until OD_600_ = 0.6, followed by induction with 1 mM IPTG. Then the temperature was lowered to 23 °C, and cells were further grown over night. The next day cells were centrifuged at 4400 × g for 30 min, and the pellet resuspended in 20 mM Tris, 100 mM NaCl, 10 mM imidazole, pH 7.8, and sonicated on ice using a Branson 450 Digital Sonifier (BRANSON Ultrasonics Corporation, USA). Cells were centrifuged at 48,000 × g for 30 min, and the presence of NTD ClpB could be verified as soluble protein on an SDS-PAGE gel. The soluble fraction was filtered through a 45 µm filter and loaded onto a HisPrep FF 16/10 nickel affinity column (GE Healthcare), equilibrated with 20 mM Tris, 100 mM NaCl, 10 mM imidazole, pH 7.8. NTD ClpB (1-156) protein was eluted using a linear gradient containing 20 mM Tris, 100 mM NaCl, 500 mM imidazole, pH 7.8. Selected fractions were dialyzed over night against 50 mM Tris, 50 mM NaCl, 1 mM DTT, 0.5 mM EDTA, pH 8.0. The His-tag was then cleaved off using TEV protease (purchased in-house from the Protein Expertise Platform at Umeå University, Sweden), added in a 1:100 (w/w) ratio, and incubated at 4 °C for 48 h. The protein was then dialyzed once more against 20 mM Tris, 100 mM NaCl, 10 mM imidazole, pH 7.8, and then loaded on the HisPrep FF 16/10 nickel affinity column, equilibrated with the same buffer. Pure NTD ClpB will pass through the column, since it is lacking the His-tag, which resulted in pure protein (SI Fig. 1A). A final dialysis step was performed to buffer exchange the protein into the proper buffer for NMR analysis (20 mM NaPi, 20 mM NaCl, pH 6.5). The final yield of pure NTD ClpB (1-156) protein was very high, where typical yields of ^15^N enriched protein reached 200 mg per liter of M9 culture.

### NMR spectroscopy

For NMR measurements, NTD ClpB (1-156) was concentrated to 1.7 mM in NMR buffer (20 mM NaPi, 20 mM NaCl at pH 6.5) and D_2_O [10% (v/v)] was added to all NMR samples for the field-frequency lock. All NMR spectra were recorded on an Avance III 850-MHz NMR spectrometer equipped with a triple-resonance cryogenic probe (Bruker, Germany). NMR spectra were processed using NMRPipe and NMRDraw software (Delaglio et al. 1995) and visualized and analyzed using CcpNMR 2.4.2 (Vranken et al. 2005). For backbone assignment, a series of triple-resonance experiments using HNCA, HN(CO)CA, CBCANH, CBCA(CO)NH, HNCO, and HN(CA)CO were performed at 310 K using uniformly [^13^C/^15^N]-labeled NTD ClpB (1-156). Assigned chemical shifts were directly referenced against 4,4-dimethyl-4-silapentane-1-sulfonic acid (DSS) for the ^1^H atoms, whereas ^13^C and ^15^N atoms were referenced indirectly as suggested (http://www.bmrb.wisc.edu). ^1^H_N_, ^15^NH, ^13^Cα, ^13^Cβ, and ^13^C chemical-shift data was used for secondary structure prediction with TALOS + (Shen et al. 2009). Secondary structure comparison was made with NTD ClpV homolog crystal structures, sequence alignments of NTD ClpB (*F. tularensis)* with NTD ClpV homologs (*E. coli* and *V. cholerae)* are shown in SI Fig. 1B.

For measurements of ^15^N-relaxation parameters, the T_1_, T_2_, T_1ρ_, and ^1^H-^15^N-heteronuclear nuclear Overhauser effects (NOEs) interleaved ^1^H-^15^ N-correlation spectra for the ^15^ N-labeled ClpB (1-156) were measured as previously described. (Dayie and Wagner 1994; Farrow et al. 1994) Backbone ^15^N T_1_ values were determined from the spectra using delay durations of 50, 100, 200, 500, 800, 1000, 1200 and 1500 ms. ^15^N T_2_ values were determined from the spectra using delay durations of 16.96, 33.92, 50.88, 84.8, 101.76, 118.72, 135.68, 152.64, 2 × 169.6, 186.56, 203.52, 220.48, 254.4, and 288.32 ms (Farrow et al. 1994), ^15^N T_1ρ_ values were determined from the spectra using spin-lock durations of 8, 24, 40, 60, 80, 120, 160, 180, 200 and 302 ms at 1.92 kHz spin-lock strength. Relaxation delays of 3 s and 2 s were used for T_1_ and T_2_ or T_1ρ_ experiments, respectively. Steady-state ^15^N-^1^H-heteronuclear NOE spectra were measured with either 5 s delays between each free-induction decay or 2 s delays, followed by a 3 s series of 120° nonselective ^1^H pulses as previously described (Dayie and Wagner 1994). T_1_, T_2_, T_1ρ_ and ^15^N-^1^H NOE experiments were performed with time-domain sizes of 256 × 2048 complex points and sweep widths of 11,029.4 and 2240.0 Hz along the ^1^H and ^15^N dimensions, respectively, with 8 scans for T_1_, T_2_ or T_1ρ_ and 24 scans for the ^15^N-^1^H NOE experiment. All ^15^N-relaxation experiments were performed at 310 K.

For ^15^N-relaxation data analysis the NMRFAM**-**SPARKY (v 1.470) (Lee et al. 2015) was used. Peak heights of the ^1^H-^15^N cross-peaks in the T_1_, T_2_ and T_1ρ_ spectra were measured using a peak-picking routine of SPARKY and fitted to a single exponential-decay function using the Curvefit module in SPARKY. Errors in T_1_, T_2_ and T_1ρ_ were estimated from the fittings using 500 Monte Carlo simulations. ^15^N-^1^H-heteronuclear NOE values were calculated from the ratio of peak intensities, *I*_*sat*_*/I*_*unsat*_, where *I*_*sat*_ and *I*_*unsat*_ represent the intensities of peaks in saturated and unsaturated spectra, respectively. ^15^N-relaxation rates were measured for the resolved and assigned resonances of the NTD ClpB (1-156). For the model-free analysis, parameters of internal motion were determined according to model-free formalism (Lipari and Szabo 1982a, b) using Modelfree4 software (v4.20; Columbia University, New York, NY, USA). Using only residues with hnNOE > 0.25, T_1_, T_2_, and NOE-relaxation data were optimized with an isotropic diffusion model using 500 Monte Carlo simulations, assuming an internuclear distance (r_NH_) of 1.02 Å and chemical-shift anisotropy of − 160 ppm for the ^15^N nucleus, generalized order parameter values were obtained (Mandel et al. 1995).

### Extent of assignments and data deposition

Backbone amide resonances in the ^1^H–^15^N HSQC spectrum of NTD ClpB (1-156) have been assigned (Fig. 1B). A total of 91% of the ^13^Cα, 86.5% of the ^13^Cβ, 87.2% of the ^13^C′ and 88.2% of the ^1^HN, ^15^N backbone resonances were assigned for NTD ClpB (1-156). The N/HN resonances were absent for residues 40–50 in the spectra, presumably due to conformational exchange or rapid solvent-exchange or both (Explanation in ^15^N backbone dynamics section). The assignments have been deposited in the BioMagResBank with accession code 51,087. These assignments of the substrate binding domain of ClpB will help to identify binding sites required for the recognition of type VI secretion sheath proteins IglA-IglB and potential drug candidates.

## Secondary structure of NTD ClpB

**Fig. 2:**
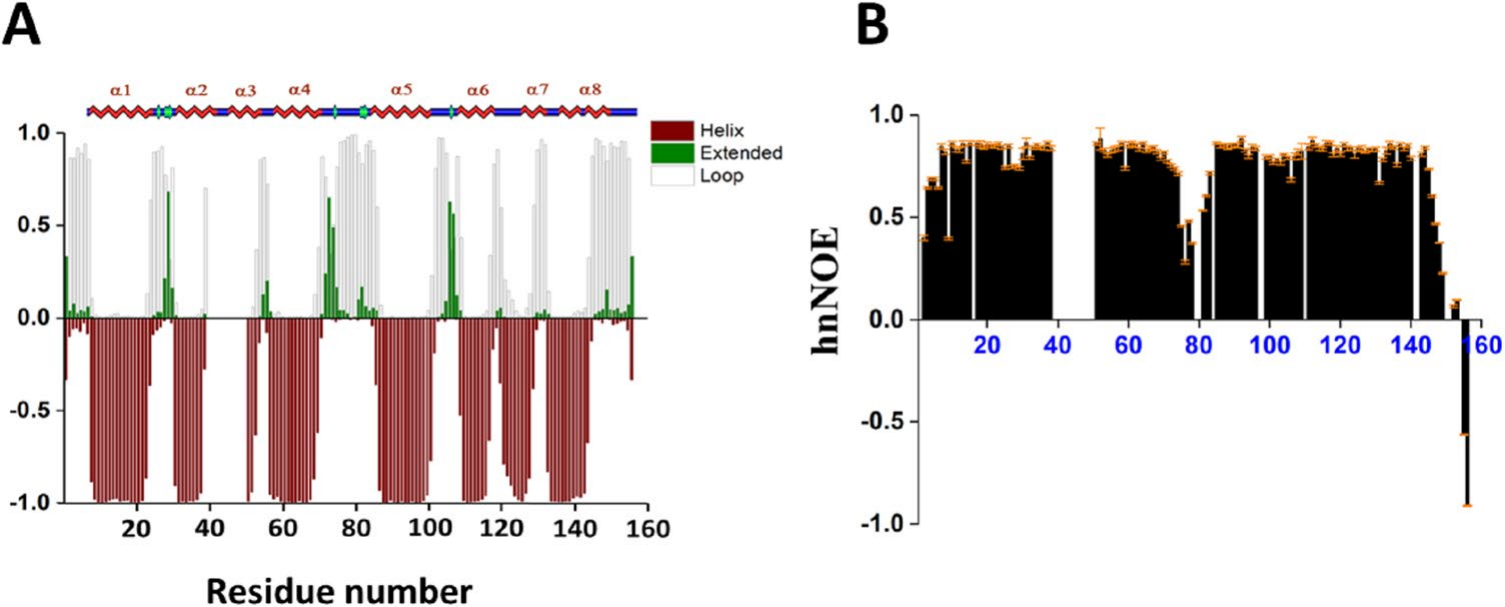
**A** TALOS + based secondary structure from assigned chemical shift data of NTD ClpB (1-156), on the top comparison is shown with the secondary structure of NTD ClpV (homolog of *E. coli*) observed in the X-ray crystal structure (PDB ID: 4HH6). Maroon, green, and gray-white bars indicate propensities for helix, β-strand, and random-coil conformations, respectively. **B** Plot of the backbone ^15^N-^1^H heteronuclear NOEs values of NTD ClpB (1-156) plotted along the residue number. Error bars are placed on top of the bar graphs

Secondary structure of NTD ClpB in solution was determined based on resonance assignments as an input in TALOS + (Shen et al. 2009). Overall the secondary structure is similar to ClpB homologs (ClpV, homolog of *E. coli*) as shown by the comparison of TALOS + based secondary structure of NTD ClpB with the secondary structure based on the crystal structure of the NTD ClpB homolog ClpV(PDB ID: 4HH6) as shown in (Fig. 2A).

### ^15^N backbone dynamics of NTD ClpB (1-156)

**Fig. 3:**
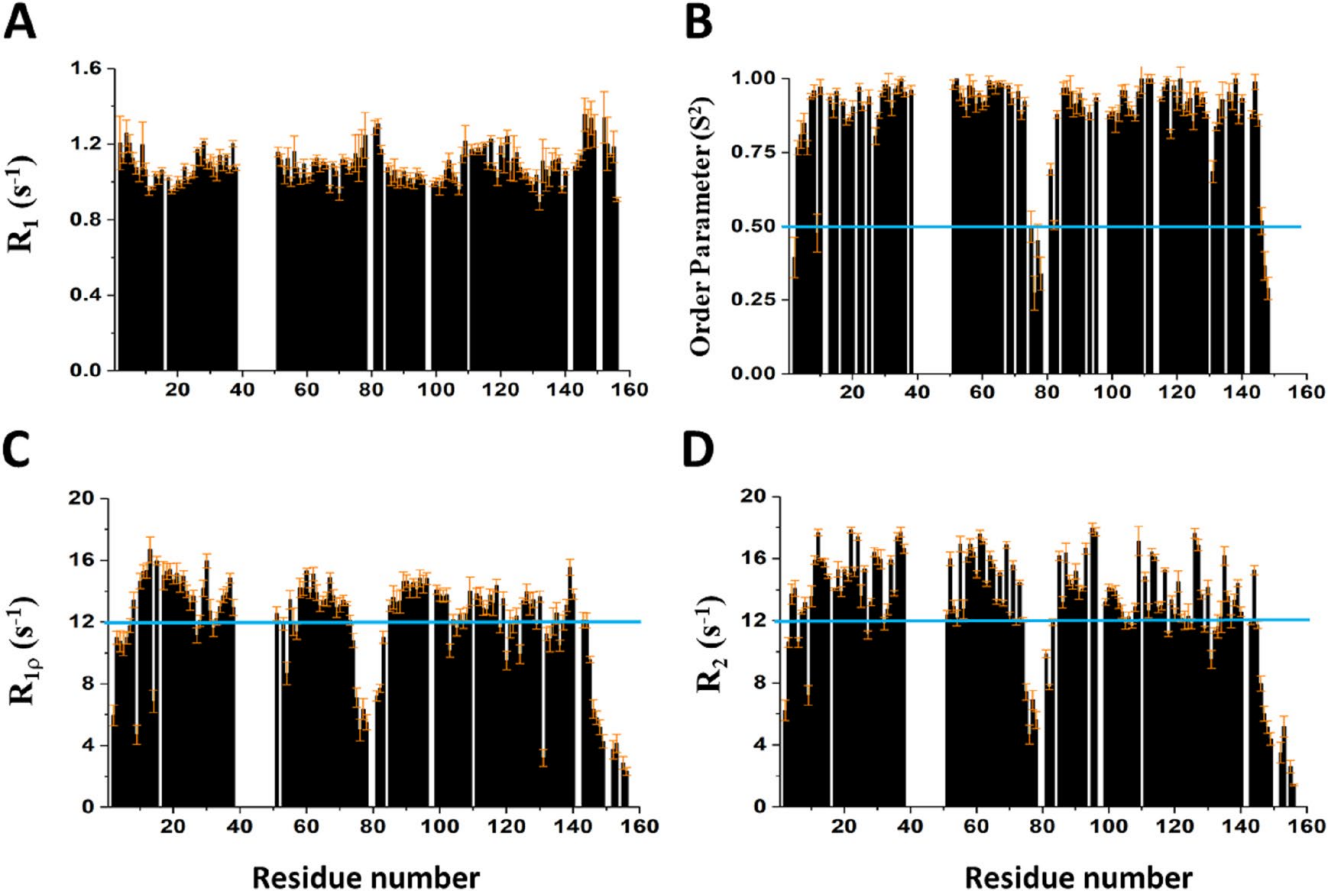
Plots of the backbone **A** R_1_, **B** Order parameter (S^2^) showing picosecond-to-nanosecond time-scale motions of the backbone amide NH vectors, **C** R_1ρ_ and **D** R_2_ values of NTD ClpB (1-156) plotted along the residue number. Error bars are placed on top of the bar graphs. All ^15^N relaxation data were acquired at 310 K and 850 MHz ^1^H frequency

We identified the flexible regions of the substrate binding NTD ClpB (1-156) using ^15^N backbone relaxation experiments. Figure 2B shows the lower heteronuclear NOE values for residues 75–82 which correspond to the loop region in ClpB homologs. The overall correlation time of NTD ClpB (1-156) was 7.52 ns at 310 K calculated from the R_2_/R_1_ ratios assuming an isotropic diffusion tensor (Kay et al. 1989). Backbone NH dynamics of NTD ClpB (1-156) were also characterized in terms of generalized order parameter (S^2^) values using the model-free formalism (Lipari and Szabo 1982a, b) with the assumption of isotropic rotational diffusion (Fig. 3D). R_2_ and R_1_ values are also shown (Fig. 3A, D) along the residue number. Comparison of R_1ρ_ and R_2_ values near the invisible-fragment residues 40–50 shows the contributions from exchange (Fig. 3C, D). Most backbone residues of NTD ClpB are rigid (Fig. 2B). The only flexible parts with increased amplitude of motions on the picosecond-to-nanosecond time scale were seen for residues 75–82 (which corresponds to the loop region in homologs ClpV), and also the residues from the far N-terminal and C-terminal ends of the NTD ClpB sequence (Figs. 2B, 3B) were very flexible.

## Supplementary Information

Below is the link to the electronic supplementary material.

Supplementary file1 (DOCX 407 KB)

## Data Availability

The backbone ^1^H, ^13^C, and ^15^N chemical shifts and ^15^N relaxation data have been deposited in the BioMagResBank (http://www.bmrb.wisc.edu/) under the accession number 51087.
