# Supplementary material for: Backbone chemical shift assignment and dynamics of the N-terminal domain of ClpB from Francisella tularensis type VI secretion system
Source: Biomol NMR Assign. 2022 Jan 5;16(1):75–9. doi: 10.1007/s12104-021-10062-3 (PMC9068650; doi:10.1007/s12104-021-10062-3)
Supplement: Supplementary file 1 — Supplementary file1 (DOCX 407 KB) [file 12104_2021_10062_MOESM1_ESM.docx]

**Supplementary information**

**Backbone chemical shift assignment and dynamics of the N-terminal domain of ClpB from *Francisella tularensis* type VI secretion system**

Ameeq Ul Mushtaq^1^, Jörgen Ådén^1^, Athar Alam ^2^, Anders Sjöstedt^2^ and Gerhard Gröbner^1,^*

^1^ Department of Chemistry, University of Umeå, SE -901 87, Umeå, Sweden

^2^ Department of Clinical Microbiology, University of Umeå, SE -901 87, Umeå, Sweden

* Gerhard Gröbner

**Email:**  [gerhard.grobner@chem.umu.se](mailto:gerhard.grobner@chem.umu.se)


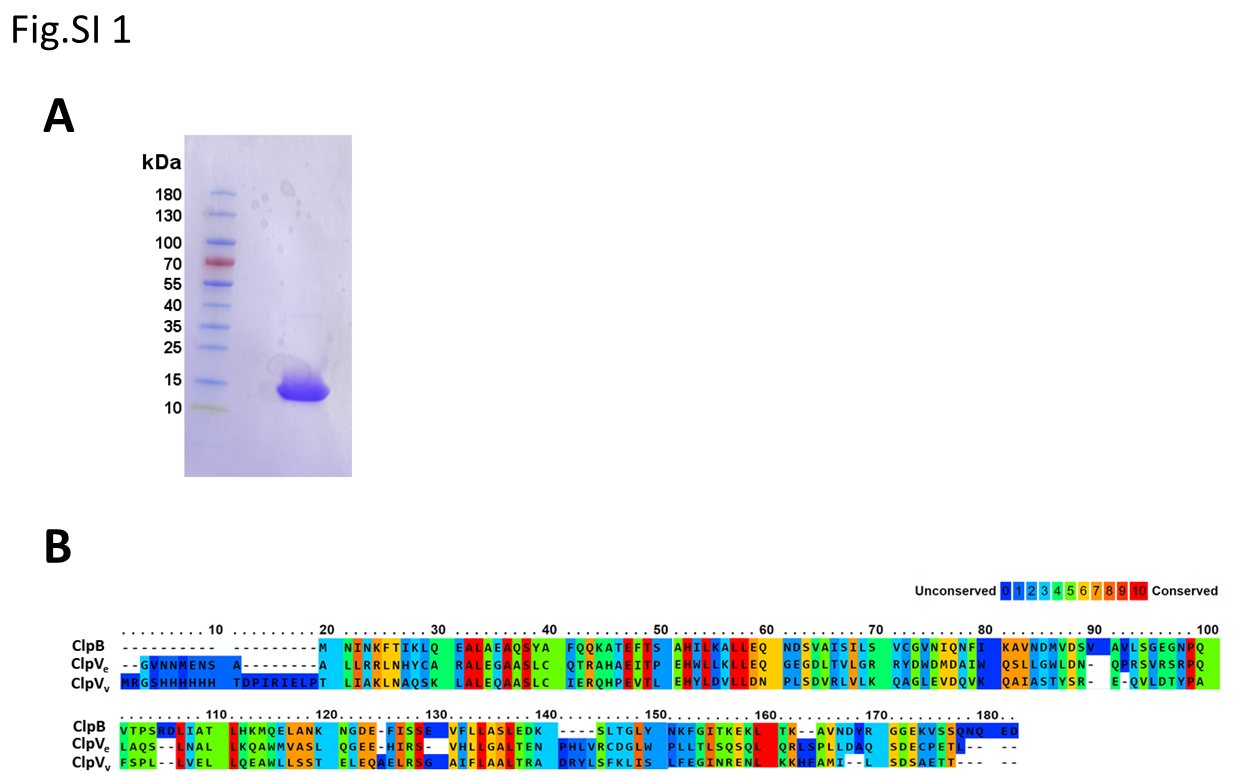


**Figure S1** (**A**) SDS-PAGE image of His-tag cleaved and pure ^15^N NTD ClpB (1-156) used for NMR analysis. Expected molecular weight of NTD ClpB (1-156) is 17.1 kDa. (**B**) Sequence alignment of NTD ClpB (1-156) sequence from *Francisella tularensis* with the NTD sequences from its close homologs ClpV_e_ (from *Escherichia coli*) and ClpV_v_ (from *Vibrio cholerae*) with known X-ray crystal structures PDB ID’s 4HH6 and 3ZRJ respectively, Colours represent the degree of homology, red blocks denote regions of sequence identity across all homologues and partially conserved residues are shown in orange to blue accordingly.
